# Engaging Stakeholders in the Development of a National Digital Mental Health Strategy: Reflexive Thematic Analysis

**DOI:** 10.2196/71601

**Published:** 2025-06-03

**Authors:** Sarah Kennedy, Robyn Fitzgerald, Ruth Melia

**Affiliations:** 1 School of Psychology Queen's University Belfast Belfast United Kingdom; 2 Psychology Department Child and Adolescent Mental Health Services Health Service Executive Limerick Ireland; 3 Psychology Department Health Research Institute University of Limerick Limerick Ireland

**Keywords:** digital mental health, policy, reflexive thematic analysis, coproduction, digital health

## Abstract

**Background:**

Recent advances in digital health technology offer the potential to overcome established access barriers to mental health support, such as stigma and geographical location. The World Health Organization recommends integrating digital technologies into mental health care, underscoring the need for countries to develop national digital mental health (DMH) strategies to guide efforts. The rate of development and availability of DMH tools currently outpaces the existing policy or regulatory guidance required to guide their use. In Ireland, a key requirement of the national mental health strategy, Sharing the Vision, was the development of a national DMH strategy. Key stakeholders in DMH research, policy, practice, and lived experience were brought together as part of a focused stakeholder engagement event to develop a shared vision for digital mental health in Ireland.

**Objective:**

This study aimed to explore the views of DMH stakeholders to set priorities for the development of a national DMH strategy.

**Methods:**

Forty-seven stakeholders were each assigned to 1 of 6 focused strategy discussion groups. Invited stakeholders included experts in DMH research, clinical practice, and mental health advocacy and policy, together with those with lived experience of accessing mental health services. Qualitative data were analyzed using a reflexive thematic analysis approach. Researchers followed the 6-step framework proposed by Braun and Clarke. Reflexive thematic analysis emphasizes intentionality and critical thought, highlighting researchers’ deliberate interpretation of data while being aware of how their perspectives shape the conclusions.

**Results:**

A total of 5 major themes were identified: inclusive access, being user-led, trust, education and training, and connectedness. These major themes were related to 15 subthemes. The inclusive access theme comprised inclusivity, accessibility, and early intervention subthemes. The user-led theme encompassed coproduction, choice, and needs-led subthemes. Compelling narrative; regulation, policy, and governance; and evidence base subthemes were identified within the theme of trust. The subthemes of digital literacy, mental health literacy, and transformation were identified within the education and training theme. Finally, the connectedness theme incorporated the subthemes of integration, relationships, and stigma.

**Conclusions:**

Overall, delegates viewed digital technology as a potential enabler of accessible and inclusive mental health support. However, it was also seen as a potential barrier to access and inclusion if concerns regarding data privacy, education and training needs, regulation, and the need for a more a robust evidence base were not addressed. Coproduction at all stages was identified as key to reducing access barriers, enhancing inclusion, and maintaining trust. Themes identified informed a follow-on consensus-seeking process to further refine and prioritize the proposed actions of the first national DMH strategy.

## Introduction

### Background

According to the World Health Organization [1], the number of people living with anxiety and depressive disorders rose significantly during the COVID-19 pandemic. Initial estimates indicate a 26% and 28% increase, respectively, for anxiety and major depressive disorders in just 1 year. While effective prevention and treatment options exist, most people with mental health difficulties do not have access to effective care [2]. Crucially, 75% of mental health difficulties are reported to have their onset before the age of 25 years [3]. The mental health of young people is an area of heightened concern and focus, driven by rising trends of earlier onset of mental health difficulties [4]. Internationally, help seeking, mental health service use, and related costs are increasing [4].

Recent advances in digital health technology offer the potential to overcome established access barriers to mental health support, such as stigma [5] and geographical location [6]. Digital mental health (DMH) refers to technology-enabled provision of mental health supports and involves leveraging the internet and related technologies, such as smartphone apps, websites, and social media, to deliver mental health services. The current ubiquitous nature of mobile phone ownership, particularly among young people [7], highlights the potential for digital technology to transform mental health service access and delivery. To achieve this, DMH interventions and plans for their implementation must be designed with end users in mind [8].

Researchers have evaluated the acceptability [9,10], feasibility [11], and efficacy of DMH interventions delivered using differing modalities [11,12] and with a broad range of participant groups [12]. A recent meta-analysis [11] indicated that web-based programs (32.3%), videoconferencing platforms (24.6%), smartphone apps (21.5%), and SMS text messaging (7.7%) were the main techniques used. Psychotherapy (67.7%) was the most commonly used intervention, followed by psychoeducation (6/65, 9%) and psychological support (5/65, 8%). Concerns remain regarding user adherence and engagement [13], the need for digital infrastructure to support equity of access [14,15], and the speed of development of DMH supports outpacing the evaluative research required to ensure quality and safety. Researchers have called for further research examining longitudinal outcomes, evidence-informed regulation and governance, and greater integration of DMH within traditional mental health services [11].

With an increase in the number of DMH supports available, researchers and advocacy groups have argued for services that are accessible to all those who stand to benefit from them [16,17]. Digital inclusion refers to the activities necessary to ensure that all individuals and communities, including the most disadvantaged, have access to and use technologies that may benefit their health [14]. Coproduction has emerged as a way to ensure that user needs are at the center of DMH design and development processes [18,19] and that the knowledge of those with lived experience meaningfully informs that process. Coproduction is critical to ensuring that policies are designed according to the needs and requirements of all involved individuals and sectors at all levels. Stakeholder engagement processes that support meaningful engagement of those with lived experience of mental health difficulties at all stages are an important part of coproduction.

DMH research, policy, and practice are also informed by established theoretical perspectives. Self-determination theory (SDT) posits that humans have 3 basic psychological needs, namely, autonomy (ie, engaging in a behavior with a full sense of volition), competence (ie, the experience of mastery and efficacy), and relatedness (ie, the need to feel connected to other people in a meaningful way) [20]. According to SDT, these basic needs are considered essential for individuals’ adjustment, integrity, and growth [21,22]. SDT postulates that need-supportive social environments improve humans’ internal motivational sources and well-being. In contrast, need-depriving (disregard for the needs) and need-thwarting (active undermining of the needs) social environments impact humans’ external motivational sources with maladaptive consequences, such as passivity and ill-being [20,22]. Thus, the social environments described earlier occupy opposite ends of a continuum that set the stage for human motivation. SDT suggests that the extent of need-support, -deprivation, and -thwarting impacts the quality of motivation (ie, the relative quantity of different behavioral regulations) [23]. SDT [24] offers a framework for understanding how digitally enabled support can influence the 3 core psychological needs—competence, autonomy, and relatedness—to promote greater engagement with services and increase the likelihood of positive outcomes [25].

The World Health Organization Global Health Strategy on Digital Health 2020-2025 [2] aims to stimulate and support every country to own, adapt, and strengthen its digital health strategy and to support countries to implement appropriate digital technologies to address their health priorities. Central to this is placing people at the center of digital health, advancing inclusive approaches, and adopting digital health technologies to scale-up and strengthen health service delivery. There have been significant and recent strategic drivers for the integration of digital technology in health services in Ireland.

The Sláintecare Action Plan [26] aimed to reform health and social care services in Ireland, over a 10-year period, espousing the need for an integrated model of care, including the use of eHealth. The national mental health strategy, Sharing the Vision: A Mental Health Policy for Everyone [27], recommends leveraging digital technology to maximize resources and promote mental health at a population level. Specifically, recommendation 31 proposes to “develop the potential for digital health solutions to enhance service delivery and empower service users.”

The publication Connecting for Life, Ireland’s National Strategy to Reduce Suicide 2015-2020 [28] identified the need to leverage digital technology to “deliver accessible information on all mental health services and access/referral mechanisms.” eHealth Ireland published the Health Service Executive National Telehealth Roadmap in 2023, which sets out a vision for telehealth in Ireland [29]: “To seamlessly integrate telehealth into business-as-usual within the healthcare service, providing high quality and safe healthcare, accessible to all, no matter who they are or where they live.”

The topics of digital health and digital inclusion are also key to the Department of Public Expenditure National Development Plan (NDP) Delivery and Reform’s Digital for Good: Ireland’s Digital Inclusion Roadmap [15].

While DMH has garnered strong policy support, ethical concerns have been raised related to insufficient effectiveness [30], data privacy vulnerabilities of DMH products [31], and a lack of adequate clinical validation and user-centered design [32]. Clear strategic guidance has been called for to help address these concerns, informed by the evidence base. However, the rate of development of DMH solutions has occurred at a faster pace than the evaluative research needed to inform such guidance. As a result, commissioning by mental health services of DMH tools can occur in reaction to increases in demand, availability in the market, or the requests of those accessing the service. However, implementation of DMH at scale is hampered by the need for a clear strategy that reflects a shared vision of DMH and addresses concerns such as quality, safety, regulation, and governance.

### Objectives

It was in this context that the stakeholder engagement process was undertaken. The primary aim was to bring together experts from research, policy, practice, and lived experience to develop a shared vision for DMH and to set priorities for the development of a national DMH strategy.

## Methods

### Context

A key requirement in Sharing the Vision: A Mental Health Policy for Everyone, Ireland’s mental health policy [27], is the development of a national DMH strategy. The DMH Specialist group was established as part of the Sharing the Vision implementation plan and was tasked with developing the strategy. The group reports to the National Implementation Monitoring Committee. The DMH Specialist group includes clinicians, mental health researchers, and patient and mental health advocacy representatives. Members appointed to the group were drawn from statutory, voluntary or charitable organizations, advocacy organizations, and research institutions (universities). It is in the context of this task that an event was organized to bring together key stakeholders to inform the development of the strategy.

### Design

Data collection occurred across three stages: (1) an open-ended survey question at registration, (2) recorded and transcribed conference presentations, and (3) facilitated focus groups.

### Participants

The conference team, with representation across research, clinical practice, and lived experience, agreed with the recruitment strategy. In the first instance, members of the DMH Specialist group were invited to attend and to put forward potential additional invitees. Members of the DMH research exchange—a network of researchers in DMH across Ireland (both Northern Ireland and the Republic)—were invited to attend and encouraged to extend the invitation to their team members. Health and allied health care professional bodies nationally were contacted, and a representative was invited to attend. Representatives from mental health voluntary or charitable organizations, mental health recovery colleges, and mental health advocacy organizations were invited to attend. Mental health service managers (HMs) across community health organizations nationally were invited to attend. Lived experience members of the conference committee invited individuals within their networks. Those tasked with mental health policy development and implementation in the Health Service and the Department of Health were invited to attend. Invitees were selected to be representative across disciplines and professional backgrounds and offer their expertise in many specialized and general fields of DMH, including clinical practice, software development and engineering, mental health advocacy, health service management and administration, mental health research, DMH co-design and development, mental health policy development, evidence synthesis, regulation, and implementation science. The overall group was gender balanced and included individuals with professional backgrounds in psychology, psychiatry, nursing, occupational therapy, physiotherapy, social work, engineering, software development, data analytics, and artificial intelligence (AI).

Delegates were primarily based in Ireland, with the exception of 2 international speakers who were invited to share their experiences as leaders in DMH internationally. One international speaker was a professor in electronic mental health with more than 20 years of DMH research experience. The second international speaker was a national lead for public mental health and a recognized leader and advocate of public mental health internationally.

Due to the public policy–focused nature of the event and the parameters of the funding award used to support the event, representatives from commercial organizations were not invited to attend.

In total, 54 participants attended. Of the 54 participants, 16 (30%) were researchers affiliated with academic institutions, 10 (18%) were clinicians employed in the health service, 7 (13%) were HMs or administrators, 9 (17%) represented mental health voluntary or charitable organizations (organization chief executive officer or designate), 4 (7%) worked in policy development (digital health, mental health, and suicide prevention), 7 (13%) identified as having lived experience of mental health difficulties or as mental health advocates, and 2 (4%) worked in recovery education. Some participants identified as having multiple affiliations. For example, of the 54 participants, 6 (11%) identified as clinicians and researchers. Certain areas of interest were more represented, such as suicide prevention, with 11% (6/54) of the attendees working in suicide prevention research, policy, training, or as suicide prevention resource officers. Of the 16 researchers in attendance, 8 (50%) were affiliated with psychology departments, 6 (37%) with computer science and engineering, and 3 (19%) specialized in AI or machine learning applied to mental health research. Of the 54 registered attendees, 48 (89%) provided a keyword or phrase at registration, 8 (15%) contributed as speakers, 4 (7%) as panel discussion contributors, and 47 (87%) took part in the focused discussion session across 6 groups.

Speakers and panel members included DMH researchers (4/16, 25%), voluntary organization chief executive officers (2/16, 12%), members of research funding organizations (1/16, 6%), health service senior management officials (1/16, 6%), policy development personnel (1/16, 6%), lived experience or advocacy representatives (2/16, 12%), recovery education personnel (2/16, 25%), and clinicians (3/16, 19%) with some speakers working across these areas. Of the 16 panel members and speakers, 8 (50%) were women and 8 (50%) were men.

### Setting

The event took place at the University of Limerick, Ireland, on November 23, 2023. Registration occurred on the web. In total, 7 conference speakers presented in person, 1 speaker presented online, and focused discussion sessions occurred on site. Each speaker presented for 10 to 15 minutes, and the focused parallel group discussion lasted 50 minutes.

### Ethical Considerations

Ethics approval was sought and received from the University of Limerick Ethics Committee (project ID 10_09_2023 EHS). Participants provided consent to share relevant data during the web-based registration process. Participants were reassured of their right to take part in the stakeholder engagement event without having their data collected for research purposes. Participants were not compensated financially for taking part in the study. Data were anonymized ahead of data analysis and manuscript submission.

### Data Collection

In the first instance, invited attendees and speakers were asked to provide a word or phrase to summarize their vision for DMH upon registering for the conference. This question was intended as a prompt and to guide attendees’ attention to the focus of the event, ahead of more detailed questions put forward within the strategy discussion session.

Second, conference speakers were asked to identify 2 to 3 words or phrases that addressed the question “A vision for digital mental health: what should it look like?” They were then invited to draw on their research, practice, or lived experience to provide a rationale for their vision during the presentation sessions.

The panel discussion members were asked to reflect on key barriers and enablers to realizing a vision for DMH and to provide concrete examples from experience that had worked well.

Facilitated strategy discussion groups, structured using agreed question prompts, invited delegates to consider the development of a DMH strategy and discuss what the vision, principles, scope, and proposed outcomes of that strategy might be (refer to Multimedia Appendix 1 for the facilitator prompts). Question prompts were informed by research, practice, and existing policy needs and were developed in consultation with a subgroup drawn from research, clinical practice, and policy. A digital recording artist captured the proceedings and discussion throughout the day. All presentations were video recorded and transcribed for further clarity. Each group was facilitated by a conference committee member, and responses were recorded by an allocated scribe.

Data collection procedures were chosen as most appropriate to explore the views of experts from diverse fields, formulate broad strategic priorities, and identify general areas of challenge and uncertainty.

### Data Analysis

The qualitative data collected were analyzed using reflexive thematic analysis. Two researchers separately analyzed all transcripts using the approach proposed by Braun and Clarke [33]. Reflexive thematic analysis involves the identification and reporting of patterns in a dataset, which are then interpreted for their inherent meaning [33-35]; these patterns can be recognized by analyzing the meaning of keywords used by participants. The 6-step framework proposed by Braun and Clarke [33] involves the following steps: become familiar with the data, generate initial codes, search for themes, review themes, define themes, and write-up. Reflexive thematic analysis emphasizes intentionality and critical thought, emphasizing the researcher’s deliberate interpretation of the data while remaining aware of how their own perspectives influence the conclusions.

In total, 2 researchers independently became familiar with the data and generated initial codes. Both researchers then met with the third researcher to discuss the codes. In the second step, both researchers began to search for the data to identify related codes and began to identify themes. Again, the research team met to discuss initial themes, identify reflective quotes, and discuss nuance in the data. In the next analytic step, data were broken down further to identify specific subthemes. The research team met again to discuss the subthemes and to discuss convergence or divergence in the data. In the final step, the researchers, using the broad themes and subthemes structure, identified opposing views, tensions, and divergence in the data and drew on reflective quotes to give voice to such data. The themes and subthemes are used as headlines in the presentation of results, and quotes from participants are included to further illustrate the views of participants.

Finally, the pillar integration process [36], a joint display technique, was used to integrate data from the initial survey question at registration, the speaker presentations, and the focused discussion sessions. To build the pillar, the researchers compared the findings that had been developed from the listing, matching, and checking stages and conceptualized the insights identified from connecting and integrating the qualitative data.

### Quality

Bryman [37] put forward strategies to improve quality and rigor in qualitative research, which the research team incorporated into the data analytic process, such as having multiple individuals code the data and then explore intercoder comparison [38]. Research team discussion was used to ensure that quotation selection would reflect robust patterns within the data and incorporate a diverse range of participants’ voices, thus ensuring an inclusive representation [39]. Researchers were also encouraged to practice reflexivity by continually examining their beliefs, values, and assumptions throughout the research process [40]. This was addressed through reflection at research group meetings and within research supervision to bring to awareness, perspectives which may impact the data analysis process. The participant descriptor key described in Textbox 1 was developed to provide a broad description of contributors’ background or affiliation, to be provided after each attributed quote in the Results section.

**Participant descriptor key.**

- RA: researchers affiliated with an academic institution
- CH: clinicians within the health service
- VC: representative from a voluntary or charitable organization
- LE: attendee with lived experience
- PDP: policy development personnel
- HM: health service manager
- RE: recovery education personnel

## Results

### Overview

Initial data collection at registration required participants to provide a word or phrase to describe their vision of DMH. Results are presented in Textbox 2. Second, speakers were invited to present on their research, clinical practice, or lived experience knowledge relevant to DMH and to provide a word or phrase to summarize their vision for DMH based on their expertise and experience (Textbox 3).

**Words or phrases provided by participants to describe their vision for digital mental health.**

**Words or phrases**

- Accessible
- Collaboration
- Connection
- Coproduced
- Efficient
- Empowering
- Transformative
- Human and responsive
- Inclusive
- Machine learning
- Mental well-being
- Opportunity
- Optimism
- Personalized
- Trusted
- Trauma informed
- Unlimited

**Words or phrases provided by speakers to describe their vision for digital mental health.**

**Word or phrase**

- Mental health literacy
- Inclusion
- Integration
- Trust and evidence
- Coproduction
- Infrastructure and support

Finally, 47 participants were divided and took part in 6 focused discussion groups in parallel, and the reflexive thematic analysis applied to the qualitative data collected resulted in the identification of 5 main themes, related to 15 subthemes. An overview of themes and subthemes is provided in Textbox 4. The themes included were inclusive access, being user-led, trust, education and training, and connectedness. The inclusive access theme comprised inclusivity, accessibility, and early intervention subthemes. The user-led theme included coproduction, choice, and needs-led subthemes. The trust theme incorporated compelling narrative; regulation, policy, and governance; and evidence base subthemes. The education and training theme related to digital literacy, mental health literacy, and transformation subthemes. The connectedness theme encompassed integration, relationships, and stigma subthemes.

**Themes and subthemes identified from focus group discussions.**

**Theme: inclusive access**

- Subtheme 1: inclusivity
- Subtheme 2: accessibility
- Subtheme 3: early intervention

**Theme: being user-led**

- Subtheme 1: coproduction
- Subtheme 2: choice
- Subtheme 3: needs-led

**Theme: trust**

- Subtheme 1: compelling narrative
- Subtheme 2: regulation, policy, and governance
- Subtheme 3: evidence base

**Theme: education and training**

- Subtheme 1: digital literacy
- Subtheme 2: mental health literacy
- Subtheme 3: transformation

**Theme: connectedness**

- Subtheme 1: integration
- Subtheme 2: relationships
- Subtheme 3: stigma

### Theme 1: Inclusive Access

#### Overview

Inclusive access was identified as a prominent theme, comprising the subthemes inclusivity, accessibility, and early intervention. Inclusive access, in this context, encapsulates the importance of broadening the reach of DMH to enhance accessibility for all. Participants detailed the multitude of ways in which DMH could facilitate greater access to mental health support. However, participants consistently highlighted the need for this access to extend to everyone. Moreover, participants argued that inclusivity is pivotal in enhancing mental health outcomes, particularly for underserved groups or groups considered marginalized.

#### Subtheme: Inclusivity

Inclusivity, as described by participants, reflects the practical ideology that DMH should be “inclusive of everybody and user friendly” (representative from a voluntary or charitable organization; VC). In reference to inclusivity, 1 participant explained the following:

If you don’t have that, there are more drop offs, especially for older and neurodivergent people.

clinicians within the health service, CH

Another emphasized that it is essential to include *“*those seldom heard voices who communicate without words, including for example, people with autism, intellectual disability or locked in syndrome, DMH strategy might consider multi-media engagement (i.e. a range of inputs/technology)” (CH).

This quote alludes to participants’ views that a DMH strategy needs to consider the expectation that modern technology automatically improves inclusivity versus the practical reality, whereby certain groups may experience additional barriers to access due to a lack of consideration of their specific needs in the design process and in the development of training. This was a consistent tension in the data, whereby digital was seen as a potential enabler or barrier to inclusivity.

There was a clear consensus that “the assumption that people understand is a barrier” (attendee with lived experience; LE). Furthermore, “it is important in the digital space, that we are using the languages that people are familiar with and can understand” (CH). An attendee with lived experience argued that jargon and acronyms complicate and reduce a mental health service user’s understanding, acting as a barrier to inclusion. One participant noted the following:

We must use the language that a specific community uses.

LE

Similarly, equity of access across geographical, societal, and digital barriers was raised by many participants:

Making sure that people have access to the hardware or devices...necessary to access it and I think that infrastructure should be taken care of from a public health perspective.

HM

Attendees noted the importance of equitable access to devices, network connection, and the broader infrastructure needed to ensure equitable access.

#### Subtheme: Accessibility

Accessibility appeared frequently, referring both to the potential for DMH to enable accessibility and the importance of designing, developing, and implementing solutions that enhance access. Practical examples of professionals’ lived experience emphasized the importance of accessibility when disseminating clinical information and designing effective treatment strategies.

However, some participants provided insights that gave a more nuanced picture of accessibility in practice. For example, 1 clinician stressed that when developing a DMH strategy, we need to “be careful we are not making another hoop for people to jump through to access services” (in the context that people with moderate-severe presentations often access several services before reaching a service appropriate to their needs).

Another participant noted that the strategy needs to incorporate wider and contextual variables that act as barriers to accessible care:

Everyone talks about online therapy during COVID but what actually came back in one area is psychologists don’t have Wi-Fi in the office and they are not prepared to risk letting a client down...they aren’t allowed to work from home to get Wi-Fi, this type of structural stuff is not working.

researchers affiliated with an academic institution; RA

The infrastructural requirements (hardware, software, and internet connectivity) were raised as potential key barriers relevant to both the accessibility and inclusivity subthemes.

#### Subtheme: Early Intervention

There was general agreement on the need for DMH to improve responsiveness and address barriers to early intervention.

One participant described how they “would love on a population level if people didn’t believe there was no access to support. People believe waiting lists are too long and there’s no point” (CH).

Some participants highlighted that digital technology has the potential to maximize clinician time and facilitate early intervention:

If some clients’ needs can be met via Digital Mental Health it may free up therapist time to give more in-person input to another client.

LE

### Theme 2: Being User-Led

#### Overview

The concept and importance of DMH strategy being user-led was frequently stressed by participants. This theme encompasses the following subthemes: coproduction, choice, and needs-led.

#### Subtheme: Coproduction

Participants highlighted the importance of implementing a national DMH strategy that is coproduced. Participants argued that central to the development of a strategy is communication with and integration of those with lived experience in the development of services:

Under that principal of co-production...there has to be an identified need, you need a leader, but you also need relevant stakeholders, family, mental health staff, community staff.

recovery education personnel; RE

Participants argued that DMH strategy development needs to involve all key stakeholders and that it is not exclusive to service users or clinicians, both of whom are key consumers of digital health. Some participants argued that integrating those with lived experience as key stakeholders not only yields positive outcomes but also brings “a level of accountability”:

[The] feedback principal of co-production-if you don’t have all the stakeholders in the room from the outset you will never get there.

RE

One participant commented on witnessing the positive outcomes of coproduction and noted that standardizing assessment tools across sites was 1 way to achieve this goal:

There is an aging research centre in...that I worked with, and it was my first time seeing real co-production of projects, service users engaging in policy and seeing changes. It came down to using the same assessment tools so they could compare across sites.

CH

#### Subtheme: Needs-Led

Several participants noted the importance of meeting *“*people where they are at” (LE) and that the implementation of digital health care should be needs-led rather than driven by the available technology. Furthermore, participants reiterated that, regardless of its dynamic nature, technology should be used as an “adjunct” to treatment, not as a replacement:

We cannot be led by the technology that is available, we have to be led by the needs of people rather than retrofitting into a piece of technology that fits it.

RE

By developing a timely strategy that incorporates the principles of coproduction and considers the needs of mental health service users, it was argued that strategy developers can alleviate the risk of being market led or driven by the technology available. However, to do so “positive risk taking has to be included in this, otherwise it won’t be user led. People need the services now” (VC).

#### Subtheme: Choice

Choice, in this context, refers to offering a broader range of mental health service delivery options whereby a person has the option of engaging with mental health support in different formats (digital or face-to-face) based on their individual preferences. Participants highlighted that to keep person-centered practice at the forefront of service delivery, those accessing mental health supports must have the choice to engage in services that best suit their individual needs:

[The] choice of delivery of intervention remains for the service user—person centred practice remains at the core.

VC

Lack of choice, or if a person feels they are being pushed toward a particular method of service delivery, may exacerbate feelings of frustration with services:

People who wanted face-to-face and when there is only online options makes them feel very isolated.

LE

One participant highlighted that ideally, they “would like a corporate structure that allows people to always have a choice to choose technology rather than they have to or there is nothing else” (CH).

### Theme 3: Trust

#### Overview

Trust was identified as a central theme consisting of the following subthemes: compelling narrative; evidence base; and regulation, policy, and governance.

#### Subtheme: Compelling Narrative

Participants reported that creating a compelling narrative in relation to DMH is essential. Participants noted that the public’s perception of DMH may be negative, as some individuals may perceive digital health interventions as a subordinate solution to face-to-face treatment or simply as a method of alleviating strain on services:

Public trust—people need to feel safe. This shouldn’t be used as a way to move people off waiting lists- [there is a] perception that this is what digital health is used for.

CH

#### Subtheme: Evidence Base

Participants highlighted the uncertainty and skepticism that comes with accessing digital resources that are not supported with good quality evidence or where the efficacy of a particular digital solution is uncertain. For instance, participants discussed young people engaging in help-seeking behavior on the web:

We now know most young people will google things when help seeking, so how do we know that they’re not being scammed?

policy development personnel; PDP

Participants noted that to alleviate these doubts, the DMH services offered “need to be robust and evidence based*”* (VC).

Some hesitancy around the lack of human involvement in certain processes was also noted. For example, some participants stated that they felt that AI could be used to a certain point; however, ultimately, there was a need for human involvement in decisions regarding health care:

If you use AI as part of anything, you use it to a point. It is part of GDPR that if you are making a decision about someone’s healthcare, you have to have a human involved, not just AI.

PDP

#### Subtheme: Regulation, Policy, and Governance

The theme of regulation, policy, and governance arose on multiple occasions among the participants who stated “regulation is important, there is science but there is also the law” (HM).

Participants noted the necessity of having regulation, policy, and governance in place, regardless of interventions themselves being evidence based, and highlighted concerns in relation to data privacy, ethical considerations, and the quality and safety of clinical practice.

Data privacy concerns were raised by participants in different contexts and in relation to various aspects of DMH. Ensuring users are fully aware of what happens to any data collected during DMH service delivery was highlighted as important in promoting trust:

In terms of the variety of technological uses, software programs to interpret that people consent to the full use of their information and what happens and that they are aware of that.

RA

Participants highlighted that standardized regulation in DMH held the potential to address concerns regarding data privacy and the quality and safety of DMH interventions:

This is where policy has to be correct as if you don’t have that it’s a free for all.

LE

Participants noted that a lack of standardization of practices and regulation brings about feelings of uncertainty and hesitation, particularly in relation to the safety of DMH interventions. Participants voiced that, above all, the priority of clinicians is to implement good, safe practice that will avoid causing harm to their clients. Other views in relation to regulation were also shared:

Regulation is obviously a positive thing, but sometimes it can limit. Which is what we’ve seen in this space so far...It boils down to being “safe,” to doing no harm.

PDP

### Theme 4: Education and Training

#### Overview

Another major theme identified in the transcripts was education and training. Participants highlighted the need for increased inclusion of DMH content within professional training programs, in continued professional development for practicing clinicians, and for those accessing DMH services and their family members or supporters. Participants also drew attention to the opportunities offered by digital technology to share knowledge and information:

Education promotion is a key piece, often people don’t realise there are digital interventions available.

RE

In turn, awareness of resources and interventions available allows for enhanced service delivery for individuals accessing health services:

Another common thing when people engage with us in education is that they leave with a sense that they are not alone.

RE

Within the theme of education and training, the subthemes of digital literacy, mental health literacy, and transformation were identified.

#### Subtheme: Digital Literacy

Participants noted the importance of introducing the concept of DMH at an early stage and “increasing awareness and Digital Mental Health training in doctoral, counselling, masters training programmes” (PDP).

Participants also reported on the importance of staff being engaged and comfortable with DMH. They noted the necessity of “implementing digital literacy as well for staff, [as] often staff [are] assumed to be on board when they may not be, service users in contrast can push for it” (CH).

In this regard, participants noted the need for specific training in relation to the integration of digital technology:

I think there’s a whole pile on education, even if tablets are issued they need to be supported with education. Some staff are not comfortable with digital and that transfers.

RE

Participants also highlighted that a lack of understanding is a barrier not only for clinicians but also for individuals seeking mental health services. Participants argued that DMH must be accurately promoted, with tailored messaging and support to ensure that individuals considering it as a treatment option fully understand what it entails:

The assumption that people understand is a barrier...in brain injury services we use the word cognitive skills and people did not understand that, so we had to rearrange all our leaflets...

CH

#### Subtheme: Mental Health Literacy

Mental health literacy was argued by participants to be important to the implementation of DMH. Participants argued that individuals are unlikely to choose to engage with DMH interventions if they are unaware of or uninformed about the evidence supporting their effectiveness compared to face-to-face support:

There needs to be more information on the effectiveness [of DMH interventions] in comparison to face to face, someone waiting a long time on a waiting list then getting online therapy they may be unhappy with this because they see it as less effective.

CH

Furthermore, participants reported that mental health literacy allows individuals to access services appropriate to them, based on their identified needs. The potentially empowering impact of having a better understanding of mental health and knowledge of services was also put forward by participants. This also underscored a need, referenced by participants, to present mental health information in a way that goes beyond a biological model of mental health and provides information and support informed by a broader psychosocial framework. In both the awareness of mental health difficulties and the evidence base relating to appropriate interventions, the provision of information was seen as empowering, providing the opportunity to transform how mental health is conceptualized and supported at an individual and population level:

There needs to be understanding between poor mental health, mental distress or mental illness because what happens is [people who have experienced] adverse childhood events access services that they should never have been assigned to according to the medical model and they end up feeling disenfranchised.

RA

#### Subtheme: Transformative

Participants highlighted the potentially transformative nature of DMH and the need for policy, education, and training to keep pace with that impending transformation:

Digital Mental Health is a rapidly expanding field, there will be huge advances in the lifespan of the policy and this needs to be accounted for.

VC

Participants note that by educating younger generations now, we can potentially alleviate further lack of understanding in the future:

If we think again about cohorts of people. Generation alpha – if we do something now, this is going to get easier over time. There are some groups we need to get up to speed, but others who won’t need to be. Thinking about Ireland in 10 years, it will be very different. If we get these basic pieces done now we will gain momentum.

VC

Participants also acknowledged that younger generations are often targeted based on the assumption that this cohort engages in a heightened use of technology and digital devices. However, many participants highlighted that digital interventions should be inclusive and target everyone, regardless of their perceived ability to engage:

What mental health services and support should it address? We focus on youth today as we assume they will interact, but naturally this will continue as people get older...ultimately it should address everyone.

VC

### Theme 5: Connectedness

#### Overview

Connectedness refers both to the interpersonal enablers of emotional connectedness and the ways in which connecting people to the appropriate services digitally can reduce stigma. This theme encompassed the subthemes of integration of services, relationships, and stigma.

#### Subtheme: Integration of Services

Participants noted that an advantage of DMH is that it can support people to connect with appropriate services, allowing for timely and responsive care. In addition, the integration of digital services with standard services expands the reach of health care professionals so people “leave with a sense that they are not alone” (LE).

However, ethical concerns arose when discussing the risk of integrating more digital services because “in the wider narrative, DMH has to be an adjunct intervention. We can’t lose sight of the fact that we are human beings” (LE).

#### Subtheme: Relationships

Participants discussed the therapeutic relationship in the context of DMH. Some participants noted the usefulness of digital technology at different stages in the relationship:

If you are already established with your clinician...the choice to do stuff online with an existing client is valuable but for someone who has never had contact it is not as valuable.

CH

As highlighted by this quote, a blended model of delivery was viewed as a means to improve adherence and independence. Specifically, this may better fit mental health service users’ needs when “moving towards discharge or reviews at that stage sometimes a text or phone check-in is easier when the relationship has already been established” (CH).

However, it was again reiterated by another participant that digitally-delivered services are “not a replacement for person-to-person connection” (VC)*.* Participants felt the benefits of virtual services are largely rooted in a preexisting face-to-face therapeutic relationship, and integrating technology, such as video-enabled care, was best considered once rapport had been established.

#### Subtheme: Stigma

The drive for connectedness also arose in the context of stigma. Participants noted that some people prefer to connect using digital tools as a means of overcoming stigma. For instance, 1 participant noted the following:

There are a certain cohort of people who would prefer a Chabot to a person, because there is no judgement.

VC

Stigma acts as a significant barrier when it comes to service engagement because “people don’t want to be seen in their community as if they have a problem with mental health” [VC].

## Discussion

### Principal Findings

#### Overview

Overall, delegates viewed digital technology as a potential enabler of accessible, inclusive mental health support and noted that actively embedding coproduction throughout the process was central to realizing this. Barriers to inclusive access included the need for appropriate digital infrastructure, education and training, a strong evidence base and research frameworks, and robust regulation and governance structures to address data privacy and other concerns. Five key themes were identified as pertinent to the development of a DMH strategy: (1) inclusive access, including subthemes of inclusivity, accessibility, and early intervention; (2) being user-led, including subthemes of coproduction, needs-led, and choice; (3) trust, including subthemes of compelling narrative, an evidence base, and regulation, policy, and governance; (4) education and training, including subthemes of digital literacy, mental health literacy, and transformation; and (5) connectedness, including subthemes of integration of services, relationships, and stigma. Analysis of these 5 themes revealed a pattern of recommendations to guide the next stage—a consensus-seeking process aimed at identifying concrete actions and priorities for the national strategy and informing implementation planning.

#### Inclusive Access

The need to address barriers and inequalities that create a “digital divide,” preventing groups considered disadvantaged from accessing DMH due to lack of access to devices, network coverage, and concerns regarding privacy and confidentiality, was highlighted consistently. Those experiencing both mental health inequalities and digital divide barriers are at a particular disadvantage, with several groups considered to be at risk of this “double jeopardy” [14]. Participants indicated that digital technology could act as a barrier to both access and inclusion if key concerns regarding infrastructure, education and training, and equity of access are not meaningfully addressed. The overarching question discussed repeatedly by participants was whether digital technology will enable greater access and inclusion to mental health support, which seemed to be met with conflicting viewpoints, ultimately indicating “that’s up to us.” The necessity for policy guidance, alongside the design, development, and evaluation process of DMH tools, to actively work to address barriers to access and inclusion was shared among participants. Participants noted inclusive access as an essential component of quality mental health services and viewed digital technology as a potential key enabler of that. Participants advocated for co-designed and user-friendly digital tools accessible to all populations, including underserved communities and groups or communities and groups considered marginalized. It was argued that this can be achieved by integrating diverse multimedia and avoiding technology that inadvertently excludes certain groups [17,18]. The findings of this study build on previous research by highlighting accessibility as a key benefit of DMH, while further emphasizing the importance of ensuring that access is inclusive for everyone. Purposefully leveraging the opportunities provided by digital technology to reach underserved groups that tend to be underrepresented in traditional mental health services was consistently argued for.

#### Being User-Led

The importance of a user-led approach in DMH was frequently advocated for by participants. This supports previous research indicating that engaging users and stakeholders in coproduction ensures services are tailored to actual needs, improving outcomes and accountability [16]. Where inclusive access was viewed as the ideal outcome, coproduction was identified as the safety rail that will support us to achieve that goal. Furthermore, prioritizing needs-led development ensures technology complements rather than dictates treatment [19]. The theme of being user-led also highlighted the importance of mental health service users being empowered to choose their preferred mode of service delivery. In line with the values of SDT [20], offering mental health service users a choice in their treatment option may fulfill 3 psychological needs (competence, autonomy, and relatedness) to support enhanced engagement in services and improve the likelihood of fostering positive outcomes [21].

#### Trust

Trust arose in the data as fundamental to the implementation of DMH. In line with research by Lattie et al [8] and Smith et al [18], participants emphasized the need for compelling narratives as well as robust evidence to overcome skepticism and perceptions of DMH as a temporary or secondary solution.

#### Education and Training

The need for comprehensive education and training across multiple levels arose in this research. Specifically, participants argued that improving digital literacy through education and training initiatives is needed to address proficiency gaps in both clinician and mental health service user groups. This theme is consistent with previous research and policy developments [41], which outlined the need for the training and development of a sustainable infrastructure of mental health professionals. Indeed, health services, such as the National Health Service, have proposed AI and digital healthcare technologies capability frameworks to address this [42]. The findings of this study advance our knowledge further by indicating the need for a greater focus on both digital and mental health literacy at a population level.

#### Connectedness

Finally, connectedness was highlighted as a crucial aspect of DMH implementation, reflecting both the importance of human connection and the potential of digital technology to connect people and services in the face of stigma or disjointed infrastructure. Integrating digital technology to enhance rather than replace face-to-face service delivery may help alleviate stigma and isolation while also addressing individual and community needs [18]. However, as echoed by the participants and noted in previous studies, such as the one by Lattie et al [8], further research is needed to explore the impact of digital modalities within different contexts and with different groups.

### Synthesis

The implementation and availability of DMH were not seen as a goal in and of itself. Rather, participants viewed digital technology as a potential enabler of accessible and inclusive mental health support. In contrast, digital technology could also be viewed as a potential barrier to inclusive access if key concerns regarding available infrastructure, mental health and digital literacy, equity of access, and privacy are not meaningfully addressed. Participants viewed coproduction as a necessary process to ensure that access barriers are addressed and DMH realizes its potential to enhance choice and placing the person at the center of their care. Across the entire process, participants moved from initially viewing DMH and discussing it as an *intervention* (eg, specific mobile apps, websites, devices, and psychotherapeutic approaches) to DMH as an *extension* of existing mental health support (eg, video-enabled care) and to finally viewing digital technology as a *force for change* in society, with the potential to not only fundamentally change how mental health support is provided but ultimately transform how we conceptualize mental health and respond to it in society.

### Limitations

This research is subject to certain limitations, primarily the potential for sample bias. Focus groups consisted of participants who likely had a preexisting preference or interest in DMH, which led to them acquiring an expertise in the area. Their attendance may have introduced an element of self-selection, which potentially impacts the generalizability of this study’s findings to a broader audience. However, the nuance apparent in the data collected indicates that participants were equally vocal during the discussion session regarding concerns, particularly in relation to data privacy, ethical considerations, and safety in service provision. The use of focus groups, while valuable for generating rich, qualitative data, may have influenced the process due to the groupthink phenomenon [43], potentially allowing for conformity to allow dominant opinions to silence others. Furthermore, industry partners were not involved in focus group discussions due to the public policy–focused nature of the event and the parameters of the funding call used to facilitate it. However, the DMH industry itself represents key stakeholders, particularly in relation to implementation, and further consultation with this group at subsequent stages will enhance strategy development and implementation. Finally, researchers often favor other methodologies, such as Delphi studies or applying content analysis, when analyzing expert views to inform strategy. However, in this study, the research team concluded that reflexive thematic analysis would be most appropriate to explore the broad range of experiences and views of a diverse group of stakeholders and that this approach was most appropriate to the stage of strategy development. Similarly, researchers identified that a Delphi approach would be more appropriately used at a later stage, for example, during the consensus-seeking process to identify priority actions or outcomes.

### Clinical Implications and Future Directions

Ensuring inclusive access to mental health support is critical in the design, development, and implementation of DMH going forward. Platforms should prioritize coproduced, user-friendly, culturally sensitive designs to engage users effectively, regardless of their level of digital or mental health literacy, age, or demographic. Coproduction of digital solutions with stakeholders ensures that DMH enhances, rather than dictates, treatment options [8]. Offering users a choice between in-person and digital care fosters a person-centered approach and reduces feelings of isolation [44]. In addition, the findings consistently underscore the importance of face-to-face interaction for many individuals in building rapport within the therapeutic relationship. Providing the choice of blended care will facilitate many individuals to overcome access barriers, such as stigma and geographical location, ensuring that digital technology is leveraged to enhance rather than replace face-to-face service provision [44].

Education and training should focus on enhancing digital literacy across clinician and mental health service user populations to bridge proficiency gaps and improve equitable access. Policy makers and commissioners should ensure that funding and continuous education programs align and evolve with technological advancements [5].

Communication of DMH research should speak to the impact and precise utility of digital interventions in mental health for specific groups. A sustainable infrastructure of DMH researchers is needed to build on and maintain an up-to-date evidence base. Standardizing DMH terminology is also important to reduce confusion and facilitate trust.

It must also be acknowledged that recent advances in AI and its application within mental health research and practice did not garner the level of discussion or focus that may be expected in the current context. The authors have reviewed and added to the existing discussion of the methodological and ethical concerns that have arisen in the area (data biases, consent to data sharing, the need for AI explainability, and human-in-the-loop processes) [45]. The comparative shortage of data relevant to this topic may reflect the timing of this event in November 2023.

### Conclusions

Participants shared a vision of inclusive, accessible mental health supports and identified digital technology as a potentially transformative enabler of this vision. It was noted that coproduction was pivotal in ensuring that digital technology is leveraged to achieve inclusive access and to avoid the compounding of access barriers for underserved groups. Specific barriers identified included access to devices, connectivity, affordability, digital and mental health literacy, and specific communication needs. Opportunities to address these barriers included improving access for those with mobility or transportation challenges, offering services in multiple languages, ensuring culturally competent care, and reaching remote or isolated populations. The need to address key priorities regarding data privacy, education and training needs, regulation and governance, and robust research infrastructure was raised. Overall, participants’ vision was not of DMH implementation as an ideal outcome but as a potential enabler of accessible and inclusive mental health care. Whether or not DMH achieves this was viewed as being dependent on the extent to which policy, governance, and regulation can address concerns, and coproduction was viewed as a necessary process to support this. Over the course of the process, participants shifted their perspective on digital technology—from seeing it as a stand-alone intervention, to a complementary extension, and ultimately as a transformative force for societal change. Themes identified were used to guide the next stage, a consensus-seeking process, which identified concrete actions for the first national DMH strategy in Ireland, and to guide implementation planning.

## Appendix

Multimedia Appendix 1 Focus group question prompts.

## Abbreviations

AI: artificial intelligence
CH: clinicians within the health service
DMH: digital mental health
HM: health service manager
LE: attendee with lived experience
PDP: policy development personnel
RA: researchers affiliated with an academic institution
RE: recovery education personnel
SDT: self-determination theory
VC: representative from a voluntary or charitable organization

## Data Availability

The datasets generated or analyzed during this study are available from the corresponding author on reasonable request.
