# Supplementary material for: Engaging Stakeholders in the Development of a National Digital Mental Health Strategy: Reflexive Thematic Analysis
Source: J Med Internet Res. 2025 Jun 3;27:e71601. doi: 10.2196/71601 (PMC12174891; doi:10.2196/71601)
Supplement: Multimedia Appendix 1 [file jmir_v27i1e71601_app1.docx]

**Appendix 1: Facilitator Prompts**

A Vision for Digital Mental Health - Strategy Discussion Session

**Aim:** The goal of this session is to harness expert opinion across research, policy, practice and lived experience to inform and guide the Digital Mental Health Strategy: Towards 2030.

**Task:** Each group will be facilitated to discuss and agree on key aspects of this strategy based on their knowledge and experience, and perhaps informed by the information shared during the event to this point. Facilitators will ensure that each discussion point receives adequate time and will endeavour to support the inclusion of all voices and opinions of the group in the discussion. The task of scribes allocated to each group is to capture and record the full breadth of the discussion.

Question Prompts relate to key areas of the strategy: Vision, Principles, Scope, and Outcomes. There are 50 minutes allocated to this session.

**Vision:** What is your vision for digital mental health in Ireland? What should it look like?

**Principles:** What principles should underpin how we achieve that vision? Are there rules, standards, ethical principles that are important to state from the outset?

**Scope:** What should be the scope of this strategy i.e. who should it apply to? What technology should it refer to? What mental health services and support should it address?

**Outcomes:** what tangible outcomes would we like to see? Or how would we know it was working? How might we measure those outcomes?

**Appendix 2:** **Proceedings and discussion captured by a visual recording artist throughout the DMH conference in visual format**

**
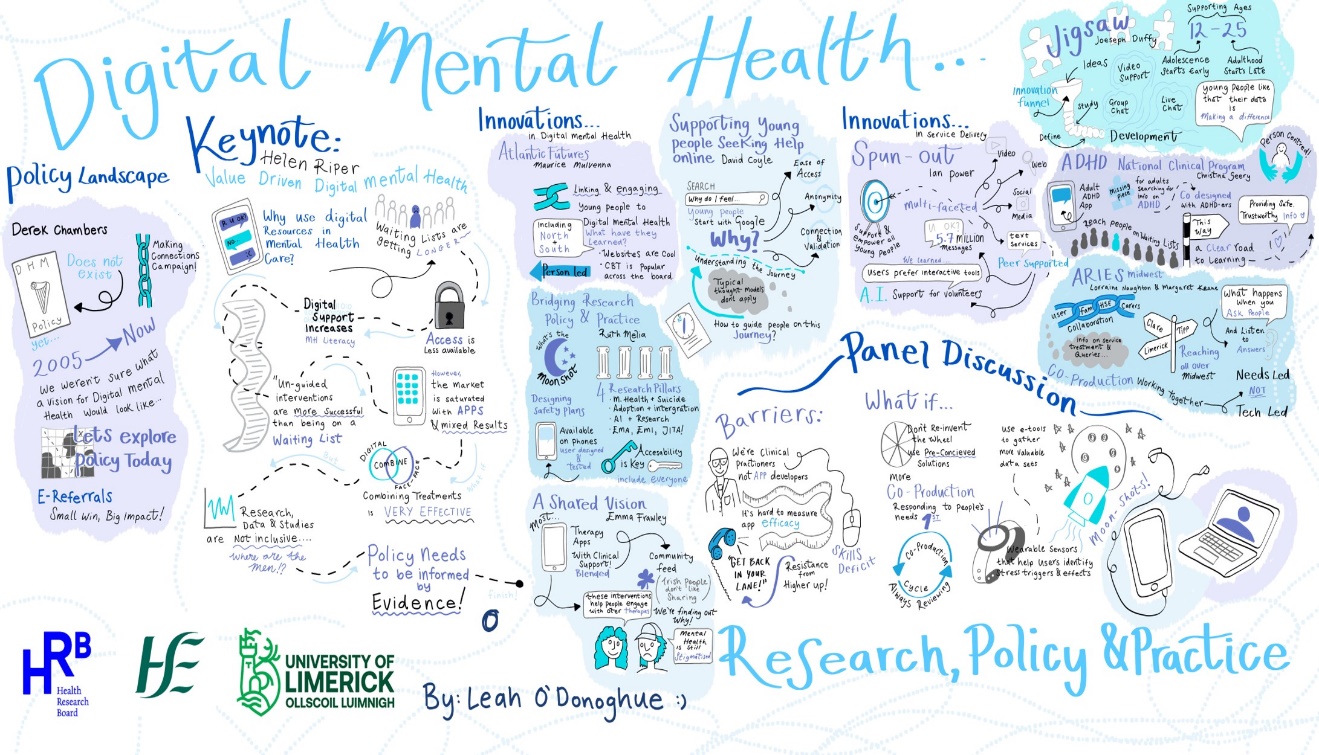
**
